# A sub-ppbv-level Acetone and Ethanol Quantum Cascade Laser Based Photoacoustic Sensor – Characterization and Multi-Component Spectra Recording in Synthetic Breath

**DOI:** 10.1016/j.pacs.2023.100473

**Published:** 2023-03-16

**Authors:** Jonas Pangerl, Elisabeth Moser, Max Müller, Stefan Weigl, Simon Jobst, Thomas Rück, Rudolf Bierl, Frank-Michael Matysik

**Affiliations:** a Sensorik-ApplikationsZentrum (SappZ), Regensburg University of Applied Sciences, 93053 Regensburg, Germany; b Institute of Analytical Chemistry, Chemo, and Biosensors, University of Regensburg, 93053 Regensburg, Germany; c Faculty of Informatics, Technical University of Munich, 85748 Garching, Germany

**Keywords:** Photoacoustic spectroscopy, Quantum cascade laser, Spectral simulation, Breath analysis, Acetone

## Abstract

Trace gas analysis in breath is challenging due to the vast number of different components. We present a highly sensitive quantum cascade laser based photoacoustic setup for breath analysis. Scanning the range between 8263 and 8270 nm with a spectral resolution of 48 pm, we are able to quantify acetone and ethanol within a typical breath matrix containing water and CO_2_. We photoacoustically acquired spectra within this region of mid-infra-red light and prove that those spectra do not suffer from non-spectral interferences. The purely additive behavior of a breath sample spectrum was verified by comparing it with the independently acquired single component spectra using Pearson and Spearman correlation coefficients. A previously presented simulation approach is improved and an error attribution study is presented. With a 3σ detection limit of 6.5 ppbv in terms of ethanol and 250 pptv regarding acetone, our system is among the best performing presented so far.

## 1 Introduction

The application of human breath analysis as a diagnostic tool is considered a major challenge. The ease of breath sampling and its non-invasive implementation is expected to reduce patient discomfort and thus enable higher patient compliance [1], [2], [3]. While there is a wide range of analytical techniques that have already been tested [1], [2], this work focuses on the laser-based sensing technique of photoacoustic spectroscopy (PAS). Compared to many other techniques, laser-based breath analysis has several advantages as it is very specific and enables fast or even real-time detection [4]. The monitoring of acetone in human breath is very desirable since acetone is a biomarker related with ketogenic diet [5], [6], [7] or acute decompensated heart failure [8]. Nevertheless, additional large-scale studies are needed to investigate the informative value of the respiratory gas composition for possible disease patterns as well as to further study the origin of acetone accumulation in human breath, as the current study situation is not conclusive [9], [10], [11], [12], [13]. A low-cost and sensitive acetone detection system could enable broad application of measurement systems and therefore large-scale studies.

PAS is a very sensitive trace gas analysis method and has great potential for miniaturization as no long absorption pathway is required [14], [15], [16]. Its application in breath analysis has been reviewed in [17], and with a focus on the near infra-red in [18], and on the mid infra-red (MIR) range in [19]. There are several wavelength regions which were explored for acetone detection covering the near infra-red (NIR) [20], [21], MIR [11], [22], [23], long-wavelength infrared [24], and even the ultra violet (UV) range [25], [26]. Viola et al. [27] and Holthoff et al. [28], for example, achieved an acetone limit of detection (LoD) of 110 ppbv (1σ) with 1 s averaging time and 555 ppbv (3σ) averaging over 3 s using an external cavity quantum cascade laser (EC-QCL) and a tunable quantum cascade laser (QCL) between 1015 and 1240 cm^−1^, respectively. Measuring acetone in breath of lung cancer patients, Mitrayana et al. [29] reported a LoD of 11 ppbv with a chopped CO_2_ laser. With a 1σ LoD of only 3 ppbv employing an EC-QCL Dunayevskiy et al. [30] obtained an excellent sensitivity. Within this work, we employed a QCL centered at 8266 nm (1209.8 cm^−1^). Centeno et al. [22] already identified ethanol to be a major interferant in this MIR region. We also investigated the influence of ethanol on our system, not only compensating for it but realizing a two-component monitoring apparatus within this spectral range of the QCL. Ethanol constitutes part of the human exhale matrix and can originate from the metabolism or through external factors like food and alcohol consumption or cleaning agents. Breath ethanol linearly correlates with blood ethanol concentration and is therefore used to assess driving ability. Together with other biomarkers, endogenous ethanol indicates the presence of the bacterium Clostridia, which is associated with pathogenic species that cause blood poisoning (e.g. tetanus) or liver diseases [31].

When a complex bulk matrix like human breath has to be analyzed, using a single specific wavelength is not sufficient, as spectral interference must be expected. To avoid incorrect concentration readings of a photoacoustic sensor, spectral PAS measurements at multiple wavelengths can be employed [11], [22], [32]. While Borisov et al. [32], [33] and Reyes-Reyes et al. [11] scanned over a large spectral range of hundreds of wavenumbers, we only investigated a very small range of 7 nm (< 1 cm^−1^), rather comparable to the work of Ciaffoni et al. [23].

A large number of biomarkers in the exhaled breath do not directly point to a distinct diagnosis of certain diseases. The occurrence of individual biomarkers rarely indicates a specific physical malfunction or disease. Most likely, several components in the exhaled air must be monitored for diagnosis. We present a QCL-PAS system to detect acetone and ethanol concentrations in a synthetic human breath^2^ matrix which is among the best performing photoacoustic acetone detection systems presented so far with a LoD (3σ) of 250 pptv with a Lock-In time constant of 10 s. The LoD (3σ) regarding ethanol is 6.5 ppbv. All measurements are carried out in the wavelength range of 8263–8270 nm with a spectral resolution of 48 pm. The QCL target wavelength was chosen upon the findings and simulattions in [38]. We further investigated typical acetone interferants, i.e., H_2_O, CO_2_ and ethanol, and verified the additive behavior of normalized single component spectra continuing our investigations presented in [34]. In addition, we improved the approach of spectra simulation published in [35].

## 2 Theory

### 2.1 Photoacoustic Spectroscopy

PAS is based on the photoacoustic (PA) effect and was first discovered by Bell in 1880 [36]. It is based on classical absorption spectroscopy, i.e., molecules absorb photons of a certain wavelength. We apply amplitude modulated PAS with a 50 % laser duty cycle allowing relaxation of excited molecules during the QCL’s off-phase. The stored energy is released as a periodic heat input causing pressure fluctuations within the medium that can be recorded as a sound signal. Through matching the laser modulation frequency with the resonance frequency of the acoustic resonator integrated into the measuring cell, a resonance-enhanced photoacoustic signal $p_{a}$ is generated. Here, the photoacoustic pressure maximum of the first longitudinal mode $f_{\mathrm{res}}$ of a tube-shaped resonator is detected by a microphone that is centered in the middle of the resonator ${\overset{⃑}{r}}_{\mathrm{mic}}$ as described in [37]. The signal can be calculated after

(1) $$p_{a}\left({\vec{r}}_{\mathrm{mic}}\right)=\underset{\mathrm{Cell}\;\mathrm{constant}\;C_{\mathrm{cell}}}{\underbrace{\left(\gamma -1\right)\frac{Q}{f_{\mathrm{res}}}\sqrt{2}\frac{L_{R}}{V_{R}}}}\varepsilon_{\mathrm{relax}}\alpha P_{0}$$

which is a product of cell constant $C_{\mathrm{cell}}$, relaxation efficiency $\epsilon_{\mathrm{relax}}$, absorption coefficient of the gas matrix α, i.e. the product of absorption cross section and analyte concentration, and the optical power $P_{0}$ of the laser at the wavelength of analyte absorption. The cell constant in turn is a linear combination of decremented heat capacity ratio $\left(\gamma -1\right)$, the ratio of quality factor Q and resonance frequency $f_{\mathrm{res}}$ as well as the ratio of resonator length $L_{R}$ and its volume $V_{R}$ normalized by $\sqrt{2}$ in terms of the first longitudinal mode of the photoacoustically generated acoustic wave. The interested reader can find a detailed derivation of the photoacoustic signal in [37], [38], [39].

### 2.2 Measures of spectral similarity

By recording the photoacoustic signal at increasing high-level currents, i.e. different discrete wavelengths due to the QCL’s tuneability, a photoacoustic spectrum can be generated. A multitude of possible quantitative similarity measures have been used. Among the most popular are the root mean squared error (RMSE), the mean absolute error (MAE) or the mean squared error (MSE) [40]. But those measures highly depend on deviations in the intensity-axis and are therefore very sensitive to a shift in the background signal. The similarity measures employed in this work are the *Pearson* and *Spearman Correlation Coefficient* (PCC and SCC). The usage of those metrics in infrared and vibrational spectroscopy is rather uncommon, albeit not new [41], [42], [43], [44], [45]. For their computation each spectrum is regarded as an intensity vector, disregarding the wavenumber information.

The PCC is computed as given in Eq. (2). It measures the linear relationship between two spectra. $x_{i}$ corresponds to the values of the intensity vector of the first spectrum, $\overset{®}{x}$ to the intensity mean, $y_{i}$ and $\overset{®}{y}$ correspond to the intensities and mean intensity of the second spectrum, respectively. A PCC value of +1 indicates a strong linear relationship, −1 an inverse linear relationship, a score of 0 no relationship. A p-value indicating the significance of the PCC can also be computed. The SCC’s computation is provided in Eq. (3), where $d_{i}$ is the difference between the ranks of $x_{i}$ and $y_{i}$ in each intensity vector. n corresponds to the number of datapoints per vector. In comparison to the PCC, the SCC is non-parametric and computed between rank variables. This makes it in general more robust towards outliers. In this work we used SciPy’s implementation of PCC and SCC (version 1.9.0) [46].

(2) $$\mathrm{PCC}=\frac{\sum_{i}\left(x_{i}-\overset{®}{x}\right)\left(y_{i}-\overset{®}{y}\right)}{\sqrt{\sum_{i}{\left(x_{i}-\overset{®}{x}\right)}^{2}\sum_{i}{\left(y_{i}-\overset{®}{y}\right)}^{2}}}$$

(3) $$\mathrm{SCC}=1-\frac{6\sum_{i}d_{i}^{2}}{n\left(n^{2}-1\right)}$$

The influence of different shapes and properties of spectra on PCC and SCC has been studied by Henschel et al. [41], [47] who conclude that the PCC is especially useful for spectra with only one very prominent feature, whereas the SCC generally achieves a higher score for multiple smaller spectral features. In addition, SCC profits from the removal of uninformative spectral regions.

Each metric has its own advantages and disadvantages. Here, the combination of SCC and PCC as a quantitative measure is preferred to typical RMSE as we expect a comparatively high systematic error in the background signal, which overly affects the RMSE measure. In addition, we underline our hypothesis with a qualitative visual analysis of our results.

### 2.3 Non-spectral interferences

Non-spectral interferences, e.g. relaxation and attenuation effects impair the simulation and evaluation of photoacoustic spectra, as they are non-linear in nature. The photoacoustic signal generation is dependent on the photoacoustic relaxation efficiency (see Eq. (1)), which can differ for every transition and depends on the bulk matrix composition [48]. This is caused by the different molecular relaxation pathways available. The inclined reader can find additional information on this challenge in [17]. To tackle the influence of changes in relaxation efficiency three different approaches exist. One is to characterize and compensate the gas matrix variations via measurements [37], [49], the other is a fully data driven approach, which applies machine learning to counter those effects building on a vast number of measurements [50], and at last a purely analytical modeling backed up by precise measurements [48]. All those approaches have their unique advantages and disadvantages. For purely analytical approaches vast literature on the gas composition in question is required, which is not available in the case of acetone and ethanol. A purely data driven approach on the other hand requires a large number of measurements to create a machine learning model. With our measurement setup the acquisition of a single spectrum takes around 3 h which renders this approach hardly feasible. To cope with those disadvantages, we apply a combination of a precise system characterization and simulation. Since the characterization of our system suggests a linear, additive behavior of the PAS signal, we apply a simplified modeling approach. We assume the photoacoustic relaxation efficiency of each infra-red (IR)-active molecule within our laser emission range to be constant and not affected by changes in the typical breath gas matrix.

Additionally, changes in the system’s resonance frequency due to bulk matrix changes can pose a problem. The system is typically specified at its resonance frequency, which changes with the speed of sound, which in turn is dependent on gas pressure, temperature, and gas matrix composition [51]. The system proposed within this work accounts for those effects by verifying the resonance frequency before and after each measurement, which will be further explained in Section 3.

### 2.4 Synthetic spectra

Synthetic spectra are very desirable in spectroscopy as they allow a low-cost data generation, which can be used to train and develop machine learning based algorithms. The feasibility of a gas detection system trained solely on synthetic data was shown by Goldschmidt et al. [52] for dual-comb spectroscopy of N_2_O and CO. Zifarelli et al. [53] use a measured dataset enriched with synthetic data to detect N_2_O and CO with an addition of C_2_H_2_ using a quartz-enhanced PAS (QEPAS) system. They apply a linear combination of their wavelength modulated measurements to augment their dataset as their spectra do not suffer from a strong background. The simulation approach reported earlier [35] and used within this work allows for the simulation of amplitude modulated synthetic PAS spectra.

## 3 Experimental

A distributed feedback (DFB) QCL from AdTech (AdTech Photonics Inc., US) together with a laser driver LDC 3736 from ILX Lightwave (Newport Corporation, US) was used for excitation between 8263 and 8270 nm (1210–1209 cm^−1^). A square modulated step signal ($f_{\mathrm{mod}}\approx 5$ kHz) provided by a frequency generator (model 33522B, Keysight Technologies, US) with a high-level amplitude between 350 and 495 mA (step width 1 mA) and a low-level current slightly below the QCL’s threshold at 250 mA was supplied to the LDC. The laser temperature was maintained constant at 23 °C. By increasing the high-level current the wavelength increased, yielding a spectral resolution of approx. 48 pm within the tuned range of approx. 7 nm (1 cm^−1^).^3^ Along with this wavelength shift, increasing the QCL current affects the optical power $P_{0}$ and, thus, the photoacoustic amplitude (see Eq. (1)) showing a second-order polynomial behaviour. Hence, it is necessary to compensate for this influence in view of the recorded spectra measurement series. As the photoacoustic background signal^4^ linearly correlates with the optical power, the scaling was carried out by taking the ratio of measured values to the respective background signal values, which were normalized to 1, i.e.,

(4) $$U_{\mathrm{PA},\mathrm{scale}}\left(I\right)=\frac{U_{\mathrm{PA},\mathrm{meas}}\left(I\right)}{b_{\mathrm{scale}}\left(I\right)}\;\mathrm{with}\;b_{\mathrm{scale}}\left(I\right)=\frac{U_{\mathrm{PA},\mathrm{BS}}\left(I\right)}{U_{\mathrm{PA},\mathrm{BS}}\left(495\mathrm{mA}\right)}\;\mathrm{and}\;b_{\mathrm{scale}}\left(I\right)\in (0;1]$$

**Fig. 1:**
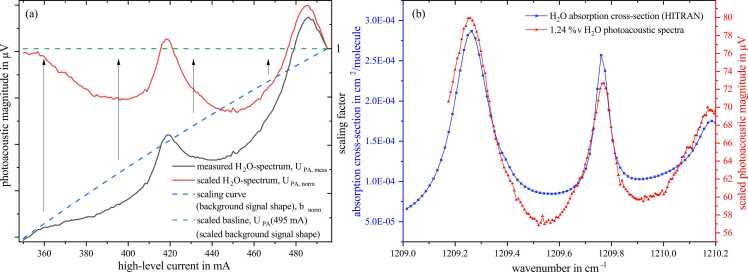
Scaling of measured spectra employing the background signal shape. In (a) the dashed lines correspond to the right y-axis. In (b) the scaled spectra of 1.24 %v H_2_O and the HITRAN absorption cross-section is visualized showing spectral concordance. Deviations in the valleys may result from fitting errors as HITRAN applies a Voigt-profile.

$U_{\mathrm{PA},\mathrm{scale}}(I)$ is the scaled photoacoustic magnitude in µV, $U_{\mathrm{PA},\mathrm{meas}}(I)$ the measured photoacoustic magnitude and $b_{\mathrm{scale}}(I)$ the scaling coefficient calculated by the ratio of photoacoustic background signal at any high level current $U_{\mathrm{PA},\mathrm{BS}}\left(I\right)$ and the background signal of the maximum high level current $U_{\mathrm{PA},\mathrm{BS}}\left(495\mathrm{mA}\right)$ (see blue dashed line, Fig. 1a). After this scaling process, every measurement point within the recorded spectrum is decoupled from the optical power. Nevertheless, the sensitivity drops with the laser current, as the PA signal voltage is simply multiplied by an individual scaling factor. Fig. 1a exemplary shows a measured water spectrum diluted in synthetic air (SA) illustrated by the black solid line. This spectrum follows the blue dashed scaling curve, which is nothing else than the background signal. Its shape is caused by the change in optical power. Dividing each point of the measured spectra with $b_{\mathrm{scale}}\left(I\right)$ yields the scaled spectrum (red solid line) which is now uncoupled from the optical power and the background signal, respectively. Fig. 1b compares the scaled H_2_O-sepctrum from (a) with the water vapor absorption cross-section provided by HITRAN verifying our measurement and scaling methods.

The photoacoustic signal $U_{\mathrm{PA},\mathrm{meas}}(I)$ detected by a microphone ICS-40720 (ICS-40720, InvenSens Inc., US) was lock-in-amplified (LIA) by an Ametek 7270 (Ametek, US) and then recorded by an in-house developed LabVIEW-based software. The photoacoutic cell (PAC) was temperature controlled at 35 °C to prevent condensation. The laboratory setup including gas delivery system as well as the PAC design have already been described in [37]. All test gases were provided by Westfahlen AG (Münster, Germany). The purity of the dilution gas synthetic air was specified with less than 0.1 ppmv of hydrocarbons. The test gases are summarized in Table 1.

**Table 1:** All gas mixtures used during the experiments excluding pure synthetic air. Westfahlen AG provided the analyte concentrations and accuracies.

Analyte gas	Buffer gas	Concentration of analyte in ppmv	Specified Accuracy in %
Acetone	SA	9.8	±5
Acetone	SA	1.2	±10
Ethanol	SA	30.5	±5
CO_2_	SA	200,000	±2

**Fig. 2:**
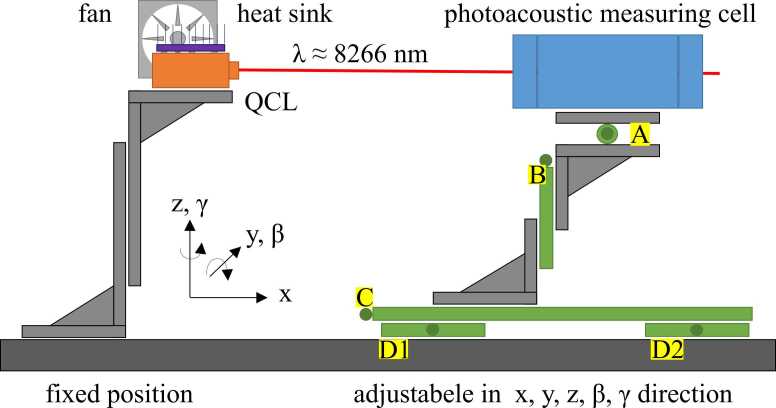
Measurement setup: The QCL combined with an active cooling system is fixed, whereas the photoacoustic measuring cell is adjustable in three translational (x, y, z) (Letters B, C, D1, D2) and two rotational directions. (β, γ) (Letter A, D1, D2).

The QCL measurement setup is shown in Fig. 2. Here, the QCL was mounted and cooled by the combination of a heat sink and an external fan to prevent the active zone from thermal destruction. Since the mounting package of the QCL already integrates a lens, no further optics are required to illuminate the PAC. The PAC was mounted on adjustable stages to optimally align the beam through the resonator, which has a diameter of 4 mm at a length of 31 mm. Using four linear stages and one rotational stage, the measuring cell and hence the resonator could be positioned and adjusted to any degree of freedom. By means of D1 and D2 the movement in the y-direction, as well as a rotation around the z-axis, could be achieved through identical or asymmetrical turning of the adjusters. This setup allows optimal alignment of the QCL-beam through the PAC without reflections at resonator walls yielding a low and stable background signal and a high sensitivity, respectively.

Additionally, we applied an acoustic resonance monitoring system (ARMS) to the PAC as explained in [54]. This ARMS is based on a speaker sweep approach yielding resonance frequency and the Q-factor determination. Such a speaker sweep was applied before and after each recorded spectrum to ensure that each measurement is performed at resonance frequency.

## 4 Results and discussion

In this work, we present the performance of a QCL photoacoustic setup for the detection of low concentrations of acetone and ethanol diluted in SA. Besides, we discuss collecting single and sum spectra with and without the analytes acetone and ethanol in the region between 8263 and 8270 nm in a typical breath matrix at ambient pressure, i.e. SA, water and carbon dioxide. The temperature of the PAC was controlled at 35 °C, the mass flow was set to 500 ml/min and the LIA roll off to 18 dB/octave, respectively. Unless mentioned otherwise, the recorded microphone signal was post-processed by (i) demodulation and low pass filtering with a time constant of $\tau_{\mathrm{LIA}}=5\;s$, (ii) data sampling with 5 Hz and (iii) averaging of the recorded points over 16 s, i.e., 80 measuring points. Finally, we report on the synthetic data generation. Here we present a comprehensive model analysis, investigating the error contribution within our system and report more on the deviations with respect to ethanol.

### 4.1 Characterization in synthetic air

The presented calibration characteristics are based on decremental analyte concentration sweeps, i.e. prediluted analyte concentrations are further diluted in SA, while the PA signal corresponding to a particular analyte concentration results from 20 s data averaging with a sampling rate of 5 Hz. The acetone concentration was varied between 9 ppmv and 1 ppmv (higher concentrated gas tank) and from 1000 ppbv to 200 ppbv (lower concentrated gas tank). The ethanol was swept from 25 ppmv to 2.5 ppmv. The measurements were carried out at ambient pressure. After the lowest set analyte concentration pure SA was applied for verifying the background signal amplitude and for determining the noise level. According to theory (refer to Eq. (1)), the photoacoustic signal is directly proportional to the number of excited molecules and thus the concentration, yielding a linear calibration characteristic. As we applied amplitude-modulated PAS, there is also a background signal in the absence of any analyte due to losses and reflections at windows and tolerances in resonator geometry and its surface quality. Table 2 summarizes some calibration and performance characteristics of our photoacoustic sensor for the analytes acetone and ethanol measured at a high-level current of 490 mA (1209.22 cm^−1^), namely sensitivity, linearity (R²), detection limit LoD (3σ), noise level 3σ, as well as the normalized noise equivalent absorption coefficient (NNEA), which is determined after [55].

(5) $$\mathrm{NNEA}=\frac{\mathrm{LoD}\;N_{A}\;\sigma \left({\overset{∼}{\nu }}_{\mathrm{Ph}}\right)\;P_{0}}{\mathrm{SNR}\;\sqrt{∆f}V_{\mathrm{mol}}}$$

**Table 2:** Summary of calibration characteristics for acetone and ethanol at a LIA time constant of $\tau_{\mathrm{LIA}}$= 5 s and 10 s.

Analyte	$\tau_{LIA}$ in s	Sensitivity in µV/ppmv	R^2^	LoD (3σ) in ppbv	Noise level (3σ) in nV	NNEA in W cm^−1^Hz^−0.5^
Acetone	5	64.1	0.99996	1.45	93.2	1.31E-9
	10			0.25	15.9	3.27E-10
Ethanol	5	7.8	0.99996	11.9	133.2	1.28E-9
	10			6.47	50.5	9.77E-10

**Fig. 3:**
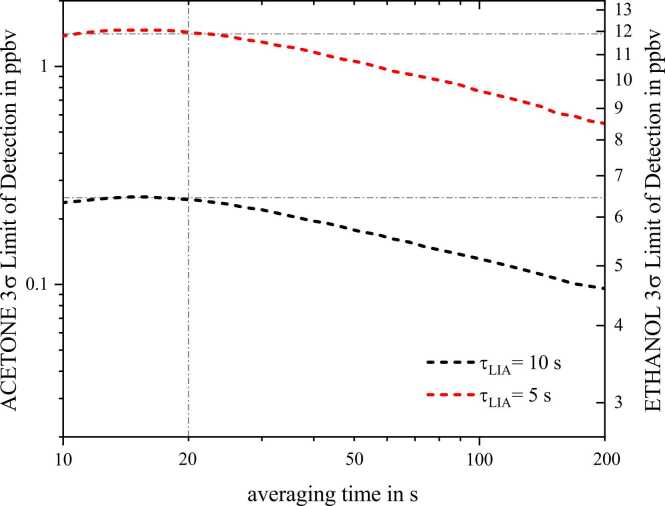
Allan Deviation plots for LIA time constant 5 s and 10 s regarding the progress of the 3σ detection limits of acetone and ethanol. The dash-dot lines indicate the measured LoDs at 20 s averaging time with a sample rate of 5 Hz.

In Eq. (5), LoD is the detection limit determined at SNR-times the standard deviation, $N_{A}$ is the Avogadro constant, $V_{\mathrm{mol}}$ is the molar volume at the prevailing temperature and pressure, $\sigma ({\tilde{\nu }}_{\mathrm{Ph}})$is the absorption cross-section of the analyte at wavenumber ${\tilde{\nu }}_{\mathrm{Ph}}$ and ∆f is the equivalent noise bandwidth (ENBW) of the LIA. The SNR equals 3 since we used three times the standard deviation for calculating the LoD. The Allan Deviation plot in Fig. 3 illustrates the LoD improvements for both analytes and two different time constants of 5 s and 10 s if the time for averaging the data is extended. The $\frac{1}{\sqrt{f}}$ characteristic of LoD improvement indicates white noise to dominate the background signal. With an LoD(3σ) of 250 pptv and an NNEA of 3.3E-10 Wcm^−1^Hz^−0.5^ the presented photoacoustic acetone detection system is among the best performing presented so far. Table 3 summarizes the characteristics and the performance of photoacoustic acetone sensors. The table indicates that we even outperform powerful CO_2_-Lasers. Note that this representative selection does not claim completeness.

**Table 3:** Summary of different photoacoustic acetone sensor approaches with their respective LoD. n.a. = not available, QEPAS = Quartz-Enhanced PhotoAcoustic Spectroscopy, CEPAS = Cantilever-Enhanced PhotoAcoustic Spectroscopy.

Reference	Method	Lightsource @ Wavelength, mean optical power	LoD in ppbv	Integration time in s
This work	PAS	QCL @ 8266 nm, 80 mW	0.25	10
Tyas et al. [56]	PAS	CO2-Laser @ 9000 to 11,000 nm, 50 W	30 (n.a.)	(n.a.)
Mitrayana et al. [29]	PAS	CO2-Laser @ 9000 to 11,000 nm, (n.a)	11 (1σ)	(n.a.)
Dunayevskiy et al. [30]	PAS	EC-QCL @ 7150–7500 nm, > 200 mW	3 (1σ)	(n.a)
Viola et al. [27]	QEPAS	EC-QCL @ 7100 to 8500 nm, > 400 mW	110 (1σ)	1
Holthoff et al. [28]	PAS	QCL @ 8064 to 9852 nm, 1.3 mW (1.1 % duty cycle)	555 (3σ)	3
Suchánek et al. [57]	CEPAS	CO2-Laser @ 9000 to 11,000 nm, 0.17 – 1 W	24,800 (3σ)	0.3
Weigl et al. [58]	PAS	UV-LED @ 278 nm, 50 mW	19.6 (3σ)	10

### 4.2 Sum spectra generation (with and without analyte)

**Fig. 4:**
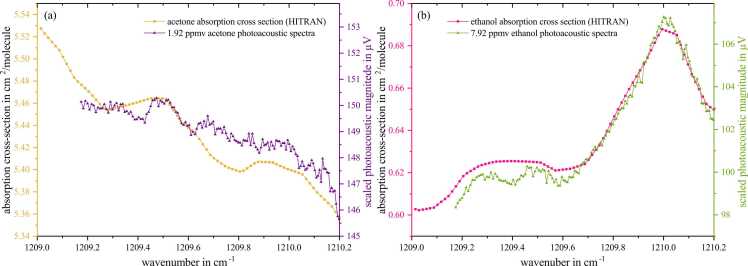
Photoacoustic spectra of 1.9 ppmv acetone (a) and 7.9 ppmv ethanol (b) together with the respective HITRAN absorption cross-section. The spectra include a constant photoacoustic background signal which is 36 µV.

The characteristics of different photoacoustically recorded spectra are investigated with respect to their behavior compared to classical absorption spectra. This involves recording spectra of only a single IR-active component (*single spectra*) as well as cumulative spectra consisting of various components, so-called *sum spectra*. Therefore, several spectra measurements were carried out based on different gas configurations of exhaled breath components. These cover measurement series with various concentrations of the analytes acetone and ethanol diluted in SA, CO_2_, and H_2_O focusing on the analyte acetone. Fig. 4 shows the scaled photoacoustic spectra of the analytes, i.e. 1.9 ppmv acetone (a) and 7.9 ppmv ethanol (b). Regarding acetone, an almost constant but slightly decreasing absorption can be observed over the full laser range, whereas a clear peak is observed in the range between 1209.7 and 1210.2 cm^−1^ in the case of ethanol measurements.

#### 4.2.1 Spectra recording with water and acetone mixtures diluted in synthetic air

**Fig. 5:**
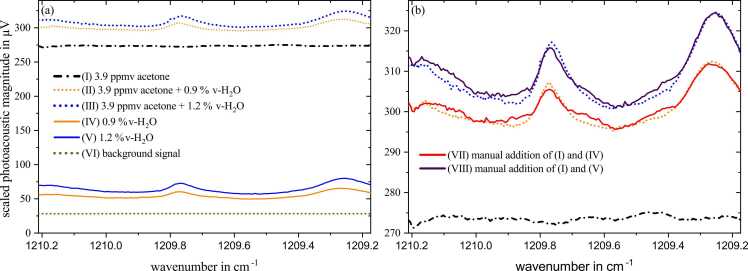
Photoacoustic spectra for different acetone and water concentrations diluted in synthetic air. (a) illustrates single spectra of one acetone (I) and two water concentrations (IV, V) as well as the measured sum spectra of two humidified acetone matrices (II, III) and the background signal (VI). In (b) the manual spectra addition (VII, VIII) of the single spectra of (a) are represented by the solid lines demonstrating an almost congruent progression to the measured sum spectra.

This section deals with the spectra recording of humidified gas samples with and without the analyte acetone. An abridged version of this topic was already published in [34]. As water is IR-active in a broad spectral range, we also found two peaks within our QCL emission range, i.e., 1209.25 cm^−1^ and 1209.77 cm^−1^. Fig. 5a presents a six spectra measurement series. The single spectra for two water concentrations, 0.9 %v and 1.2 %v, and for the acetone concentration of 3.9 ppmv are shown by the graphs IV, V, and I, respectively. Besides, the sum spectra for the mixtures of acetone and both water concentrations are illustrated by the graphs II and III as well as the background signal (graph VI) which equals a straight line due to normalization. Fig. 5b shows again a detailed view of the graphs I, II and III from 5a and furthermore the resulting graphs obtained from the manual addition of the respective single spectra, i.e. I + IV and I + V. The comparison of the measured and calculated sum spectra shows an almost congruent progression with only marginal deviations, which can be attributed to measurement inaccuracies and artifacts. To quantitatively analyse this additive characteristic of spectra, the linear correlations after Pearson and Spearman (PCC/SCC) were calculated, which reached values of 0.9831/0.9620 and 0.9855/0.9823 for 0.9 % and 1.2 %v-H_2_O, respectively. Those values are considered to indicate very good correlation. Since the multiplicative property of acetone concentration was already proven within the concentration sweep in section 3.1, the same behaviour in terms of water was verified by measuring two different water concentrations. Linearity was identified, investigating the respective spectra IV and V with respect to the background signal VI. This yielded a sensitivity of 42.7 µV/%v-H_2_O and an R^2^ of 0.9999 calculated at a high-level current of 485 mA, i.e., the local maximum of the water peak. Hence, both additive and multiplicative characteristics of single and sum spectra could be verified among measurements with acetone and water diluted in SA. This implies a constant relaxation efficiency for both single and multicomponent PAS, allowing the measured curves to be considered as real absorption spectra.

This finding allows for a straightforward separation of the two components when applying linear regression. For this approach two measurement points were taken from the constructed spectrum, one placed at a water absorption peak at 1209.31 cm^−1^ and one at 1209.52 cm^−1^ where only weak absorption from water was present. A regression fitted on the three single component measurements and the constant background (Fig. 5: I, IV, V, VI) was used to estimate the concentration of the two multi-component measurements. The regression predicted 0.96 %v and 1.21 %v-H_2_O (set values were 0.89 %v and 1.24 %v-H_2_O) and 3.92 and 3.91 ppmv acetone (set values were 3.92 ppmv acetone each) for the two measurements (Fig. 5: II, III). This prediction from only two measurement points shows how the spectral interference of water can be compensated for while at the same time quantifying the water concentration of the sample. Hence, the analyte detection is very precise with overlapping water absorption bands with a mean average percentage error (MAPE) of only 0.26 %.

#### 4.2.2 The impact of CO_2_ on spectra recordings of acetone and humidified acetone mixtures

Besides water, carbon dioxide is also present in the exhaled breath in the percentage range. This section therefore discusses the influence of CO_2_ on the photoacoustic spectra recordings originating from gas mixtures with and without humidity and analyte, respectively. In accordance with HITRAN database, no CO_2_ absorption was found experimentally within our region of laser emission. Accordingly, the shape of the spectrum does not change due to CO_2_ absorption. However, CO_2_ influences the speed of sound and therefore the resonance frequency as well as the Q-factor and the adiabatic exponent, which cause the cell constant and therefore the photoacoustic amplitude to decrease with increasing CO_2_ concentration in dry mixtures. Once water is added to a mixture containing CO_2_, this effect disappears, i.e. the amplitude is no longer decreased by CO_2_ addition.^5^ Table 4 lists measured and calculated values affecting the cell constant for different mixtures of CO_2_, H_2_O, and acetone. Since CO_2_ causes the resonance frequency to decrease by approx. 15.0 Hz/ %v-CO_2_ and water to increase $f_{\text{res}}$ only by approx. 5.4 Hz/%v-H_2_O, mixtures containing both species show a resonance frequency below that of mixtures without CO_2_. Nevertheless, the influence of the Q-factor predominatess (1 %/%v-CO_2_ in dry mixtures) compared to the change in resonance frequency. The change in the adiabatic exponent is also less significant. The relative change in cell constant calculated in the right column of Table 4 is referred to the background signal cell constant without analyte, CO_2_, and water (first line in Table 4) and provides a compensation factor with respect to the influence of water and CO_2_. Therefore, the photoacoustic amplitudes of dry measurements containing CO_2_ were compensated after

(6) $$U_{\mathrm{PA},\mathrm{comp}}=U_{\mathrm{PA}}\frac{1}{1-\left[X_{\mathrm{CO}2}\right]b_{\mathrm{CO}2}}$$

with $U_{\mathrm{PA},\mathrm{comp}}$ being the compensated photoacoustic amplitude, $\left[X_{\mathrm{CO}2}\right]$ the CO_2_ concentration in %v and $b_{\mathrm{CO}2}$ the compensation factor for dry CO_2_-measruements which was determined to 0.83 %/%v-CO_2_ with R^2^ = 0.9971. Acetone as well as ethanol do not affect $f_{\text{res}}$, Q or γ as these analytes are only present in trace concentrations.

**Table 4:** Impact of CO_2_ and H_2_O on $f_{\text{res}}$, Q and γ (with and without acetone). Values labelled in bold correspond to calculations, the others are based on experiments. The line marked with an (*) belongs to a typical synthetic breath mixture containing 500 ppbv acetone.

CO_2_ in %v	H_2_O in %v	Acetone in ppmv	f_res_ in Hz	*Q*	**γ**	**ΔC_cell__in_ %**
0	0	0	5049	37.8	**1.3995**	**reference**
0	0.89	0	5053	37.9	**1.3989**	**0.03**
0	1.24	0	5056	38.0	**1.3986**	**0.01**
0	0	3.92	5049	37.8	**1.3995**	**-0.05**
0	0.89	3.92	5053	37.9	**1.3989**	**-0.04**
0	1.24	3.92	5057	38.0	**1.3987**	**0.04**
3	0	0	5003	37.0	**1.3961**	**-2.33**
3	0.89	0	5005	38.2	**1.3954**	**0.77**
3	1.24	0	5009	38.2	**1.3952**	**0.69**
3	0	1.96	5004	37.0	**1.3961**	**-2.27**
3	0.89	1.96	5005	38.0	**1.3954**	**0.28**
3	1.24	1.96	5008	38.0	**1.3952**	**0.29**
5	0	0	4971	36.2	**1.3937**	**-4.19**
5	0.89	0	4972	38.2	**1.3931**	**0.98**
5	1.24	0	4975	38.1	**1.3928**	**0.62**
5	0	1.96	4972	36.2	**1.3938**	**-4.11**
5	0.89	1.96	4975	38.2	**1.3931**	**0.79**
5	1.24	1.96	4977	38.1	**1.3929**	**0.40**
* 4	1.5	0.5	5001	37.8	**1.3939**	**0.08**

#### 4.2.3 Spectra recording of synthetic breath

**Fig. 6:**
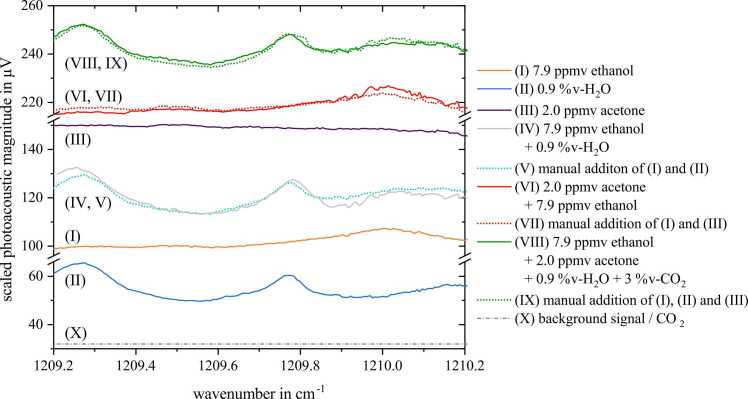
Photoacoustic spectra for ethanol and acetone together with water and CO_2_ diluted in synthetic air. Single spectra of ethanol, water and acetone are illustrated by I, II and III, the others are sum spectra and manual additions. Measured spectra are represented by solid lines, manual spectra addition with identical gas matrices are visualized by the dashed lines.

This chapter finally deals with spectra recording of all components we are currently considering with regard to breath analysis, i.e., the two analytes, acetone and ethanol, and the main components of exhaled breath, namely water, carbon dioxide, nitrogen and oxygen. Although a typical breath sample contains less analyte, we set the ethanol concentration to 7.9 ppmv, as otherwise no noticeable peak could apparently be seen in Fig. 6. Fig. 6 displays single and sum spectra of 7.9 ppmv ethanol, 2.0 ppmv acetone, 0.9 %v-H_2_O, and 3.0 %v-CO_2_ diluted in SA, respectively. As CO_2_ does not absorb within the laser emission spectrum, it does not have any spectral influence. The ethanol spectrum (I) shows a baseline of about 100 µV with a visible peak at approx. 1210.0 cm^−1^. Manual addition of (I) and the water spectrum (II) yields the sum spectra (V) of analyte and water, which was experimentally validated in (IV). The addition of the analyte spectra (I) and (III) results in the sum spectra achieved by manual addition (VII), experimentally validated in (VI). Finally, the empirically obtained sum spectrum of synthetic breath as a whole is represented by (VIII), while manual addition of individual spectra is illustrated by (IX). Pearson and Spearman correlation coefficients of measured and calculated sum spectra are summarized in Table 5. With scores well above 0.8 they are considered to match very well according to [45]. The comparatively high difference between PCC and SCC in case of the spectra VI and VII is comprehensible considering the visualized spectrum. It is still within expected bounds and the difference stems from the different spectral features both scores represent.

**Table 5:** Pearson and Spearman correlation values of measured and calculated sum spectra.

Gas mixture	PCC	SCC
IV, V	0.913	0.906
VI, VII	0.952	0.841
VIII, IX	0.969	0.968

Again, both additive and multiplicative characteristics of single and sum spectra could be verified among these measurements with synthetic breath which reinforces the assumption of constant relaxation efficiency. In terms of spectral reconstruction, i.e., both a quantification and separation of a measured sum spectrum into its individual components, the CO_2_ content can be determined via the measured resonance frequency $f_{\mathrm{res},\mathrm{meas}}$ obtained by the ARMS (refer to [54]). As the present humidity content is continuously monitored by a *pTH*-sensor (BME 680, Robert Bosch GmbH, Germany) within the measuring cell and the resonance shift coefficients of water and CO_2_ were specified above, the present CO_2_ content [$X_{\mathrm{CO}2}]$ can be recalculated via

(7) $$\left[X_{CO2}\right]=\frac{f_{\mathrm{res},\mathrm{BS}}-f_{\mathrm{res},\mathrm{meas}}-\left(\left[X_{H2O}\right]\kappa_{H2O}\right)}{\kappa_{H2O}}$$

where $f_{\mathrm{res},\mathrm{BS}}$ is the resonance frequency of the background signal (5049 Hz), $\left[X_{H2O}\right]$ the measured water concentration, $\kappa_{H2O}$ the change in resonance frequency through water addition (5.4 Hz/%v- H_2_O) and $\kappa_{\mathrm{CO}2}$ the frequency coefficient regarding CO_2_ (15.0 Hz/%v-CO_2_), respectively. This method yields a LoD(3σ) of 1.19 %v-CO_2_.

Altogether, about 30 spectra with different gas configurations were recorded. Combined with the findings that the addition of bulk components affects the photoacoustic spectra linearly, we proved our spectra not being influenced by relaxation effects. All this data, together with literature and simulation inputs, provide a data basis for a machine learning model that can quantify the individual components from the measured sum spectra similar to the linear regression presented in section 3.2.1. Additional research and effort extending from linear regression is needed, as the differentiation between ethanol and acetone in this spectral region proves more difficult due to their flat absorption profiles.

### 4.3 Spectral simulation

**Fig. 7:**
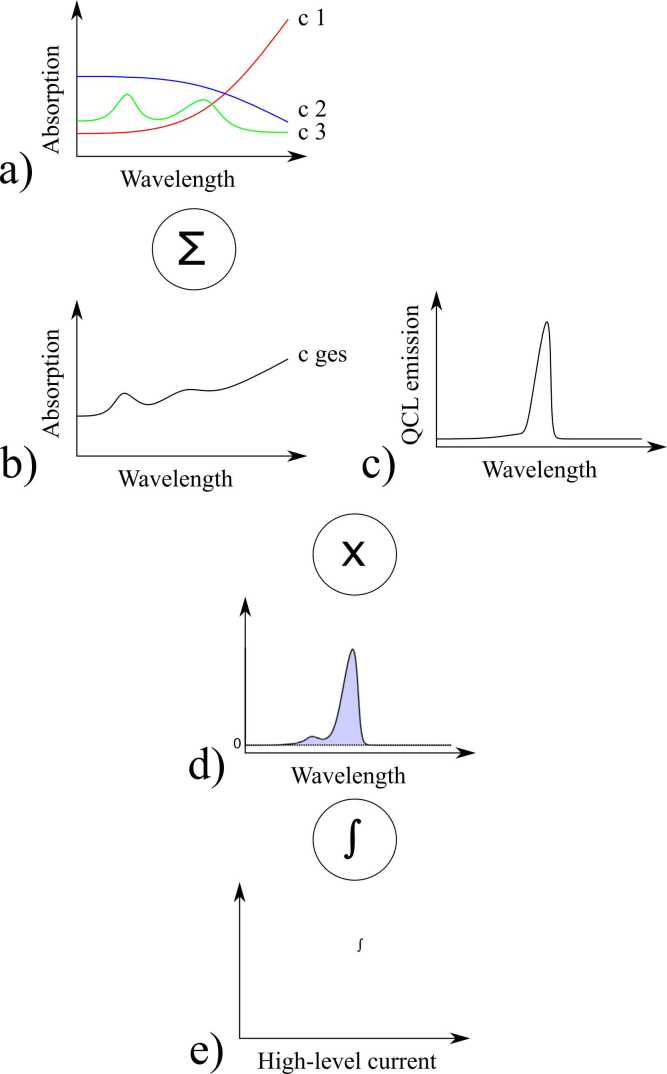
Partial workflow of the simulation algorithm. The scaled absorption spectra from HITRAN or PNNL databases (a) are summed (b). The QCL output spectrum of a certain high-level current (c) is multiplied with the summed spectra (d) and the area under the curve is computed (e). This process is repeated for each high-level current and scaled with the factor q(I); the background b(I) is added to create the actual output spectrum of the system (not shown).

The analysis of our system shows a linear, additive behaviour of the investigated spectral components during characterization. This allows for the use of a simple simulation system to further investigate our results. The simulation amounts to applying an intricate instrument function, as well as scaling and an additive background signal to a summation of the absorption spectra of the components, which are available in spectral databases. A basic version of this simulation has already been presented in [35] and is visualized in Fig. 7. The basis of the simulation are absorption spectra from literature, either simulated from the HITRAN line database [59], [60] as for CO_2_ and H_2_O, or measured spectra from the PNNL database [61] as for acetone and ethanol. Those are adapted for the prospective concentration and subsequently summed. As an instrument function the laser output spectrum is convoluted with the absorption spectrum. This process is repeated for every high-level current value, the current supplied to the laser during the on phase. The resulting PAS spectrum is scaled, and the background signal magnitude is added.

(8) $$p_{a}\left(I\right)=q\left(I\right)\left[P_{\tilde{\nu }}\left(I\right)*\sum_{c}A_{\tilde{\nu }c}\right]+b\left(I\right)$$

The model equation is presented in Eq. (8). Upper-case letters indicate vectors. $p_{a}(I)$ is the measured photoacoustic magnitude at high-level current I, $q\left(I\right)$ is the scaling factor which represents a combination of photoacoustic parameters like optical power and the Q-factor. $P_{\tilde{\nu }}(I)$ represents the optical output power of the laser at high-level current I. This vector is convoluted with the summed and scaled absorption spectra. $A_{\tilde{\nu }c}$ is again a vector representing the simulated absorption spectrum of each component for the expected concentration. Finally, $b\left(I\right)$ constitutes the background signal magnitude for each high-level current I.

Only two single molecule spectra for acetone, ethanol, carbon dioxide, water, and two background measurements were used to create this simulation, which amounts to a total of 10 measurements. Within this work, a more intricate analysis of the system than the one presented in [35] is provided and suggests improvements to the simulation algorithm to account for systematic errors we discovered regarding ethanol.

#### 4.3.1 Model analysis - Error contribution

The pursued modelling approach allows for a detailed error analysis and error attribution. We present an in-depth analysis of the model presented in [35] with a quantitative error analysis and attribution study. We selected three quantities of interest for further analysis: The background signal, the amplification factor and the QCL transfer function. For those three quantities we investigated a constant component and a component that scaled linearly with the supplied high-level current. Those correspond to the original fitting parameters c_2_, c_1_, d_1_, d_0_, b_5_, and b_4_ [27, (1), (2), (3)]. There were more parameters applicable to the simulation, i.e. a quadratic background component, which were not analysed further. We computed the variance of those parameters from the covariance matrix and confirmed the feasibility of those estimates using an affine-invariant ensemble sampler for Markov Chain Monte Carlo (EMCEE) [62]. For this computation 28 measurements were used, including the 10 measurements for model creation. The results are presented in Table 6.

**Table 6:** Relative error of selected model parameters. All other parameters remained fixed during computation.

Parameter	Relative Variance
Transfer function (linear)	0.18016
Transfer function (constant)	2.0677E-05
Background signal (linear)	0.015121
Background signal (constant)	0.028315
Amplification factor (linear)	0.021535
Amplification factor (constant)	0.010959

The resulting relative errors were mostly between 1 % and 3 % which corresponds to a well-defined model. Only the parameter associated with the linear part of the QCL transfer function showed a very high error of 18 %. The constant background function parameter is constant for every high-level current within one simulation and thus corresponded to a horizontal baseline signal for a non-normalized signal. Whereas the linear background function parameter corresponded to a parameter which scaled linearly with the high-level current within one simulation and thus corresponds to a linear baseline for a non-normalized signal. The transfer function parameters on the other hand can be understood as the position of the output peak at a certain high-level current. It was modelled including a quadratic component, but the contribution was only computed for the constant and linear part. The EMCEE showed a high uncertainty of the linear parameter and high correlation with the other transfer function parameter, which probably caused the high relative error. This was expected as the optical QCL output spectra acquired with a spectrum analyzer (BRI-771-B, Bristol Instruments Inc., US) and used to generate the model had an unsatisfying resolution. Even though the other relative errors were small, the constant background error of 2.8 % corresponds to a variation in the background baseline of +/- 1.6 µV, which justified our choice of SCC and PCC over the RMSE as error measure. The low error of the amplification parameter confirmed that measurements were performed at resonance frequency. The visible changes can be linked to the influence of CO_2_ and water on the quality factor of the cell. The model is deemed acceptable for further investigation with the highest improvement potential in the laser transfer function.

#### 4.3.2 Model analysis - Systematic error with ethanol

One main advantage of a system to model photoacoustic spectra from theoretical absorption spectra is that it allows for an intricate analysis of the system, especially in the case of discrepancies between theory and measurement. We discovered some deviations with the modelling of ethanol in our case, regarding the signal height as well as its shape and have analysed their origin.

**Fig. 8:**
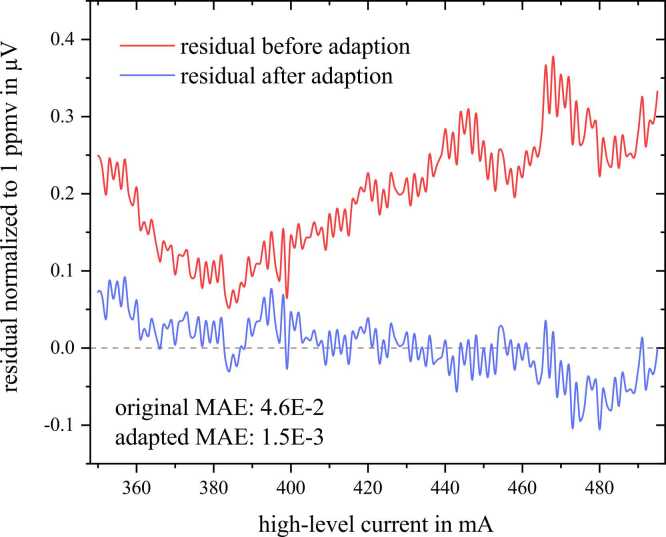
Residual error (simulation-measurement) of the absorption spectra of ethanol. Three ethanol measurements were normalized to 1 ppmv ethanol concentration, and their mean was taken.

We noted a systematic discrepancy between our modelled ethanol spectra and the measured spectra. This observation was made in the validation spectra as well as in the spectra used for model creation. In Fig. 8 the residual error is visualized dependent on the supplied high-level current (red line). We noted a systematic error in the overall simulated signal height which shows in the shift on the y-axis over all high-level currents. We traced this error back to a comparatively high concentration error in the supplied ethanol gas tank, which only contained 96.5 % of the assigned concentration. This was still in the bounds of the margins specified by the supplier (+/−5 %) but led to noticeable errors. Within the model this can be resolved either by readapting the model creation process with corrected concentration values, or as was done within this work by applying a factor of 0.965 to the ethanol base spectrum $A_{\tilde{\nu }c}$.

After this correction a discrepancy in the shape remained, which shows in Fig. 8 as a pronounced valley from 360 to 420 mA. This error can be mainly attributed to differences of the ethanol spectral shape between our measurement and PNNL spectra in the region from 8261 to 8263 nm, which influences the signal in this current range. We additionally verified that the errors did not stem from QCL output simulation and the background signal. We decided to correct this error via an adaption of the underlying ethanol base spectrum, which has been taken from PNNL [61]. We numerically adapted the PNNL base spectrum between 8260 and 8264 nm. These two adaptions reduced the MAE of the normalized ethanol spectra from 46 nV to 1.5 nV, which improved the model substantially. This results in an improved modelling of the measurements compared to the original simulation presented in [35].

## 5 Conclusion

We developed a highly sensitive photoacoustic system for detecting the biomarkers acetone and ethanol in SA and synthetic breath bulk mixtures, respectively. With a 3σ detection limit of 250 pptv for photoacoustic acetone detection we achieved, to the best of our knowledge, the lowest LoD reported so far. The ethanol limit of detection was 6.5 ppbv. Besides, we measured approx. 30 photoacoustic spectra with different gas matrixes to compare additive and multiplicative properties of those spectra to classical absorption spectra. We found our system did not suffer from any non-linear or non-spectral interferences. The PCC and SCC values for the addition of water to an acetone mixture diluted in SA proved a very good consistency of measured sum spectra and manual addition of the single spectra. The exact shape of the water peaks at 1209.25 cm^−1^ and 1209.77 cm^−1^ can be incorporated to compensate the recorded sum spectra and correctly quantify a humidified sample. Adding CO_2_ to a synthetic breath sample does not affect the absorption spectra and hence not the photoacoustic spectra within our QCL emission range. Therefore, the actual CO_2_ content can be calculated on behalf of measured coefficients for frequency detuning resulting from major buffer gas changes induced by water and CO_2_. Knowing the water concentration from an integrated humidity sensor to the photoacoustic measuring cell, the CO_2_ concentration can easily be calculated via the frequency detuning coefficients for water (+5.4 Hz/%v-H_2_O) and CO_2_ (−15.0 Hz/%-CO_2_). A photoacoustic signal deterioration in terms of a dry CO_2_ containing breath sample is not relevant for practice since already a minor humidified sample completely compensates this effect. Mixing all components of our in-lab synthetic breath, i.e., the two analytes acetone and ethanol combined with the main components of exhaled breath, namely water, carbon dioxide, nitrogen, and oxygen we again identified a linear behaviour of measured sum spectra versus the manual addition of measured single spectra. PCC and SCC verified our photoacoustic spectra recordings and computations. In consequence, these data can be used as a basis for a simulation approach. The previously proposed simulation approach was verified towards those measurements and an error attribution study was presented. The relative variance of the investigated parameters used during simulation was below 3 %. Only a parameter corresponding to laser transfer function showed a higher variance. This higher variance can be attributed to insufficient resolution in the data used to generate the model and high correlation between the three parameters used to simulate the transfer function. The proposed modelling approach was adapted with respect to major discrepancies of the ethanol base spectrum. The simulation approach verified by this study can serve as the basis towards a machine learning approach to identify and quantify an unknown breath sample of our analytes as well as CO_2_ and water concentration.

## Funding

Essential financial support for this work has been provided within the scope of the project BreathSens funded by the German Ministry of Education and Research (BMBF) with founding code 13GW0325C as well as the project PreSEDA funded by the German Federal Ministry for Economics and Climate Action (BMWK) with funding code 03EN2028A. Besides, funding was received by the BayWISS-Health and BayWISS Digitalization network financed by the Bavarian Ministry of Research and Arts. Additionally, two of the authors are funded by a PhD scholarship of the Studienstiftung des Deutschen Volkes and the Hanns-Seidel Stiftung. One author was furthermore funded by the Marianne-Plehn-Programm of the Elitenetzwerk Bayern funded by the Bavarian Ministry of Research and Arts.

## CRediT authorship contribution statement

**Jonas Pangerl:** Conceptualization, Data curation, Investigation, Methodology, Visualization, Writing original draft, Project administration, Writing – review & editing. **Elisabeth Moser:** Conceptualization, Data curation, Investigation, Methodology, Software, Writing original draft, Writing – review & editing. **Max Müller:** Conceptualization, Methodology, Validation, Writing – review & editing. **Stefan Weigl:** Conceptualization, Methodology, Validation, Funding Acquisition, Writing – review & editing. **Simon Jobst:** Conceptualization, Methodology, Validation, Writing – review & editing. **Thomas Rück:** Conceptualization, Methodology, Validation, Writing – review & editing. **Rudolf Bierl:** Funding acquisition, Project administration, Supervision, Validation, Writing – review & editing. **Frank-Michael Matysik:** Conceptualization, Methodology, Supervision, Validation, Writing – review & editing.

## Declaration of Competing Interest

The authors declare that they have no known competing financial interests or personal relationships that could have appeared to influence the work reported in this paper.

## Footnotes

2 Real breath also contains about 1 %v argon, which is ignored here as argon does not influence the photoacoustic signal within our emission wavelength range.

3 Indeed, a higher tuning range can be achieved by also varying the QCL’s temperature.

4 As we apply amplitude modulated PAS, there is an acoustic background signal mainly originating from interactions of the light beam with cell windows, e.g. absorption processes which reducess the optical power by about one third.

5 Actually, the photoacoustic magnitude slightly increases when water is added to dry CO_2_ containing mixtures compared to the background magnitude due to an increased Q-factor. The linear influence of adiabatic exponent as well as resonance frequency are of minor significance.
